# Association of Dietary Patterns with Metabolic Syndrome: Results from the Kardiovize Brno 2030 Study

**DOI:** 10.3390/nu10070898

**Published:** 2018-07-13

**Authors:** Antonella Agodi, Andrea Maugeri, Sarka Kunzova, Ondrej Sochor, Hana Bauerova, Nikola Kiacova, Martina Barchitta, Manlio Vinciguerra

**Affiliations:** 1 Department of Medical and Surgical Sciences and Advanced Technologies “GF Ingrassia”, University of Catania, via S. Sofia 87, 95123 Catania, Italy; agodia@unict.it (A.A.); andreamaugeri88@gmail.com (A.M.); martina.barchitta@unict.it (M.B.); 2 International Clinical Research Center, St Anne’s University Hospital, 656 91 Brno, Czech Republic; sarka.kunzova@fnusa.cz (S.K.); ondrej.sochor@fnusa.cz (O.S.); hana.bauerova@fnusa.cz (H.B.); n.kiacova@seznam.cz (N.K.); 3 Institute for Liver and Digestive Health, Division of Medicine, University College London (UCL), London NW3 2PF, UK

**Keywords:** diet, nutrition, metabolic disorders, obesity, hypertension, diabetes, hyperlipidemia

## Abstract

Although metabolic syndrome (MetS) could be handled by lifestyle interventions, its relationship with dietary patterns remains unclear in populations from Central Europe. Using data from the Kardiovize Brno cohort, the present study aims to identify the main dietary patterns and to evaluate their association with MetS risk in a random urban sample from Brno, Czech Republic. In a cross-sectional study of 1934 subjects aged 25–65 years (44.3% male), dietary patterns were derived by food frequency questionnaire (FFQ) administration and principal component analysis. Metabolic syndrome was defined according to the International Diabetes Federation statement. Logistic regression models were applied. High adherence to the prudent dietary pattern was associated with lower odds of abdominal obesity, abnormal glucose concentration, and MetS. By contrast, high adherence to the western dietary pattern was associated with higher odds of abnormal glucose, triglycerides and blood pressure levels. Whilst our results confirm the deleterious effect of a western dietary pattern on several metabolic risk factors, they also indicate that the consumption of a diet rich in cereals, fish, fruit and vegetables is associated with a healthier metabolic profile. However, further prospective research is warranted to develop and validate novel potential preventive strategies against MetS and its complications.

## 1. Introduction

Metabolic syndrome (MetS) is a growing public health concern worldwide, which is associated with an increased risk of cardiovascular morbidity and mortality [1,2,3,4]. Among the multiple classifications for the diagnosis of MetS, one of the most widely used definitions is that developed by the International Diabetes Federation (IDF) [5], which defines MetS as the combination of clinical and metabolic factors, including insulin resistance, hyperglycemia, hypertension, dyslipidemia and abdominal obesity [5]. MetS is frequently accompanied by lipid accumulation in the liver—this condition is also defined as nonalcoholic fatty liver disease (NAFLD)—which is considered the hepatic manifestation of MetS. Interestingly, NAFLD and metabolic syndrome overlap in many aspects, not only including the spectrum of diseases that they predict but also risk factors associated with them [6]. 

In the last decades, prevalence of MetS has dramatically increased, likely due to changes in lifestyle factors, socioeconomic status and dietary habits. Given this scenario, one of the greatest challenges in the management of MetS is to alleviate the risk associated with modifiable factors, such as obesity, physical activity and diet, through lifestyle interventions [7]. Although the association of dietary patterns with the risk of MetS has been well-established, as confirmed by recent meta-analyses [8,9], evidence substantially varied across populations. Whilst a “prudent” dietary pattern—a healthy diet characterized by high intakes of vegetables, fruits and fish—was inversely associated with MetS [10], a “western” dietary pattern—characterized by high intakes of red and processed meat, refined grains, alcohol and fried foods—increased the risk of MetS [11]. However, results from other studies were inconclusive [12,13]. This inconsistency could be attributed not only to difference in the prevalence of MetS [14], as well as in the definition used for the diagnosis [15], but also to dietary habits that vary between populations. In fact, there is considerable variation in food intakes across Europe and also within countries [16]. To explore the complex association of biological, environmental and behavioral risk factors with cardiovascular diseases (CVD), we recently designed the Kardiovize Brno 2030 study, which recruited a randomly-selected sample of residents in an urban population of Brno, Czech Republic [17]. The Kardiovize Brno 2030 study provides evidence from a European region where epidemiological studies are scarce, focusing on a population with a peculiar lifestyle: while more than half of Kardiovize subjects are highly physically active, less than five percent have a healthy diet [18]. Previous results from this population suggested that eating more frequently and consuming breakfast may be potential preventive strategies against weight gain and CVD risk [19]. In the current cross-sectional analysis, using data from the Kardiovize Brno 2030 cohort, we aim to identify the main dietary patterns and to evaluate the association of these patterns with the risk of MetS. 

## 2. Materials and Methods

### 2.1. Study Design

In this cross-sectional study, we used data from the Kardiovize Brno 2030 cohort which included a random sample of residents from the city of Brno, Czech Republic [17]. The study protocol was approved by the ethics committee of St Anne’s University Hospital, Brno, Czech Republic (reference 2 G/2012), in accordance with the Declaration of Helsinki. Data were collected and stored in the web-based research electronic data capture (REDCap) [20]. Demographics characteristics, socioeconomic status, cardiovascular risk behaviors and medical history were collected by trained professionals through a face-to-face comprehensive health interview. In the current analysis we included participants (i) of both sexes, (ii) aged 25–65 years and (iii) regardless menopause status in women, (iv) with no previous/current cardiovascular disease, (v) who had complete information on anthropometric measurements, cardio-metabolic parameters, sociodemographic, diet and lifestyle. We excluded subjects with extreme values of total energy intake (<500 or >3500 kcal) and diabetic patients, since the diagnosis of diabetes may influence their dietary habits. 

### 2.2. Dietary Assessment

Dietary data were obtained by a 43-item Food Frequency Questionnaire (FFQ) using the previous week as reference period. The intake of alcohol drinks (i.e., wine, beer, dessert wines and spirits) was assessed using beverage-specific weekly recall. For each food item, participants were asked to indicate frequency of consumption classified into seven categories, ranging from “almost never” to “six or more times a day”. Standard portion sizes—defined as the age- and sex-specific median food intake obtained from an individual dietary survey on the national level [21], which involved age and gender representative sample of the Czech population [22]—were attributed to each food item. Food intakes were derived from the FFQ by multiplying frequency of consumption by standard portion size of each food item. Food intakes were adjusted for total energy intake—assessed using the NutriDan software—by the residual method [23]. 

### 2.3. Principal Component Analysis

*A posteriori* dietary patterns were derived using principal component analysis (PCA). Food items were classified into 31 predefined food groups, based on the similarity of nutrient profiles or culinary usage. Individual food items that constituted a distinct item on their own (e.g., pasta, pizza, or eggs, etc.) or that represent a particular dietary pattern (e.g., alcoholic drinks and fries, etc.) were preserved. Factors analysis was performed on energy-adjusted intakes of each predefined food group followed by varimax rotation, which maintains uncorrelated factors facilitating the interpretability. The number of dietary patterns was defined based on eigenvalues >2.0, Scree plot examination, and interpretability. To characterize each dietary pattern we considered factor loadings with absolute value ≥0.250. To confirm internal reproducibility, we separately performed factor analysis in two randomly selected subgroups by using the same abovementioned approach. For each dietary pattern, factor scores were calculated by summing the products between observed energy-adjusted food group intakes and their factor loadings. For each dietary pattern, factor scores were categorized by tertiles (T1 = low adherence; T2 = medium adherence; T3 = high adherence); the lowest tertile (T1) of each dietary pattern was used as the reference for further analyses.

### 2.4. Anthropometric Measurements

Anthropometric measurements were performed after overnight fasting by trained researchers, according to previously described protocols [17]. Briefly, height and weight were measured to the nearest 0.5 cm and 1 kg, using a medical digital scale with meter (SECA 799; SECA, GmbH and Co. KG, Hamburg, Germany). BMI—defined as weight in kilograms divided by height in meters squared—was categorized as underweight (<18.5 kg/m^2^), normal weight (18.6–24.9 kg/m^2^), overweight (25–29.9 kg/m^2^), and obese (≥30 kg/m^2^). Waist, hip and neck circumferences were measured to the nearest 1 cm by using a manual measuring tape. Waist-to-hip ratio (WHR) was calculated by dividing the waist measurement by hip measurement. According to ethnic specific values, abdominal obesity was defined as waist circumference ≥94 cm in men and ≥80 cm in women. Body fat mass (BFM) was assessed using a direct segmental multi-frequency bioelectrical impedance analysis (InBody 370; BIOSPACE Co., Ltd., Seoul, Korea).

### 2.5. Biochemical Analyses and Physical Examination

Cardio-metabolic parameters were assessed as described elsewhere [17]. Briefly, blood pressure (BP) was measured using a mercury sphygmomanometer (Baumanometer, W.A. Baum, Co., Inc., Copiague, NY, USA). Biochemical parameters were evaluated on 12-h fasting peripheral blood samples using a Modular SWA P800 analyzer (Roche, Basel, Switzerland). Particularly, total cholesterol, triglycerides, fasting glucose and creatinine were assayed by the enzymatic colorimetric method (Roche Diagnostics GmbH, Penzberg, Germany), while HDL- and LDL-cholesterol by the homogeneous method through direct measuring without precipitation (Sekisui Medical, Tokyo, Japan). If triglyceride levels were lower than 4.5 mmol/L, we calculated LDL-cholesterol according to the Friedewald equation. 

### 2.6. Definition of Metabolic Syndrome

According to the International Diabetes Federation (IDF), we defined MetS for participants with abdominal obesity who met at least two out of four criteria: fasting glucose ≥100 mg/dL (5.6 mmol/L) or treatment for type 2 diabetes; HDL cholesterol <40 mg/dL (1.03 mmol/L) in men and <50 mg/dL (1.29 mmol/L) in women or drug treatment for lipid abnormality; TG ≥ 150 mg/dL (1.7 mmol/L) or drug treatment for lipid abnormality; and systolic BP ≥ 130 mmHg or diastolic BP ≥ 85 mmHg, or drug treatment for hypertension [5].

### 2.7. Lifestyle Factors

Physical activity level and intensity (walking, moderate, and vigorous) were assessed using the long version of the International Physical Activity Questionnaire (IPAQ-L) [24] translated in Czech, which includes four domains (leisure time, work/commuting, home and garden/yard). Physical activity was reported as Metabolic Equivalent of Task (MET-min/week) and classified as follows: high (vigorous-intensity activity on at least 3 days and accumulating at least 1500 MET-min/week or 5 or more days of any combination of walking, moderate-intensity or vigorous intensity activities achieving a minimum of at least 3000 MET-min/week); moderate (3 or more days of vigorous activity of at least 20 min per day or 5 or more days of moderate-intensity activity or walking of at least 30 min per day or 5 or more days of any combination of walking, moderate-intensity or vigorous intensity activities achieving a minimum of at least 600 MET-min/week); low (subjects who not meet criteria for categories 2 or 3) [24]. For smoking status, subjects were classified as no smokers (never being a smoker or having quit >12 months) and current smokers (daily or occasionally). 

### 2.8. Other Covariates

Face-to-face comprehensive health interviews were carried out on demographics/socioeconomic status and medical history. The following information were recorded: demographics and socioeconomic status (age, gender, educational level, and employment status); self-reported medical history (diagnosis and treatment of hypertension and hyperlipidemia). Particularly, hypertension was defined as BP greater than or equal to 140/90 mmHg, or a prior diagnosis of hypertension or taking antihypertensive drugs. Hyperlipidemia was defined as having either total cholesterol greater than or equal to 5.0 mmol/L, or LDL cholesterol greater than or equal to 3 mmol/L, or triglycerides greater than or equal to 1.7 mmol/L, or taking lipid-lowering drugs. 

### 2.9. Statistical Analyses

Statistical analyses were performed using the SPSS software (version 22.0, SPSS, Chicago, IL, USA). The Kolmogorov–Smirnov test was used to assess the normal distribution of variables. Descriptive statistics were used to characterize study participants using frequencies or median and interquartile range (IQR). Trend across tertiles of dietary patterns was analyzed using Kruskal–Wallis test for continuous variables and Chi-squared test for categorical variables. To assess the association between dietary patterns and the risk of MetS and its components, multiple unconditional regression models were applied. Odds ratios (OR) and 95% confidence intervals (CI) were calculated using the following models: model 1 adjusted for age and sex; model 2 adjusted for age, sex, marital status, employment, educational level, smoking, BMI, total energy intake, and physical activity. All statistical tests were 2-sided, and *p* values less than 0.05 were considered statistically significant.

## 3. Results

### 3.1. Study Population and Prevalence of MetS

A total of 1934 participants, aged between 25 and 65 years (mean = 46.6 years in men; mean = 47.2 years in women), were included in the current analysis. Approximately half the sample (55.7%) was female and 23.7% were current smokers. The majority of the sample was employed (82.0%) and married (61.7%). Median values of total energy intake and physical activity level were 2006 kcal (IQR = 916) and 3375 MET-min/week (IQR = 4782), respectively. Forty-nine % of subjects were overweight or obese, with 52.6% meeting or exceeding the cut-offs for abdominal obesity. The prevalence of hypertension was 34.6%, with 17% of participants receiving antihypertensive treatment. Specifically, median values of systolic and diastolic BP were 117.0 mmHg (IQR = 19.5) and 79.0 mmHg (IQR = 13.0), respectively. The prevalence of hyperlipidemia was 65.4%, with 5.4% receiving drug treatment for lipid abnormality. Specifically, median values of HDL and triglycerides were 1.52 (IQR = 0.50) and 0.99 (IQR = 0.76), respectively. Median fasting glucose level was 4.8 (IQR = 0.7), and none of the participants received antidiabetic treatment since we excluded diabetic patients from the current analysis. Based on the IDF definition, prevalence of MetS was 38.2%, with 41.0%, 15.9%, 22.0% and 9.5% of subjects who showed abnormal BP, HDL, triglycerides and fasting glucose, respectively. 

Table 1 shows the comparison of socio-demographic characteristics, anthropometric measures and cardio-metabolic parameters between MetS patients and controls. Briefly, MetS group consisted of more male (48.6% vs. 42.2%; *p* = 0.017) and older subjects (median = 54.0 years, IQR = 15.0 vs. median = 41.5 years, IQR = 17.0; *p* < 0.001), with higher percentages of low-educated (61.0% vs. 46.4%; *p* < 0.001), married (69.1% vs. 58.3%; *p* < 0.001), unemployed (25.7% vs. 13.3%; *p* < 0.001) and smoking (26.7% vs. 22.1%; *p* = 0.041) subjects. As expected, MetS patients exhibited higher anthropometric measures and cardio-metabolic parameters, but lower total energy intake, physical activity and HDL cholesterol level than controls. 

### 3.2. Dietary Patterns

**Figure 1:**
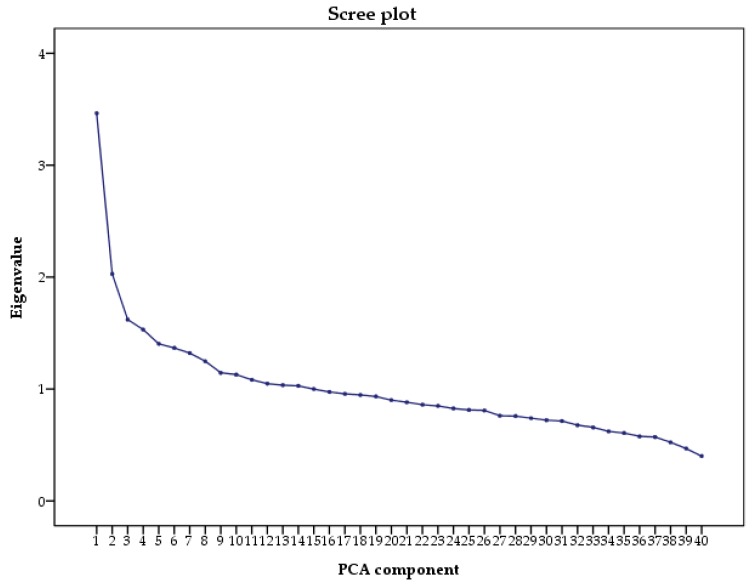
Scree plot of the eigenvalues. We used the scree plot examination to determine the appropriate number of dietary pattern. Scree plot represents the partitioning of the total variation (i.e., eigenvalue) accounted for each principal component, against the principal component number. PCA: principal component analysis.

**Figure 2:**
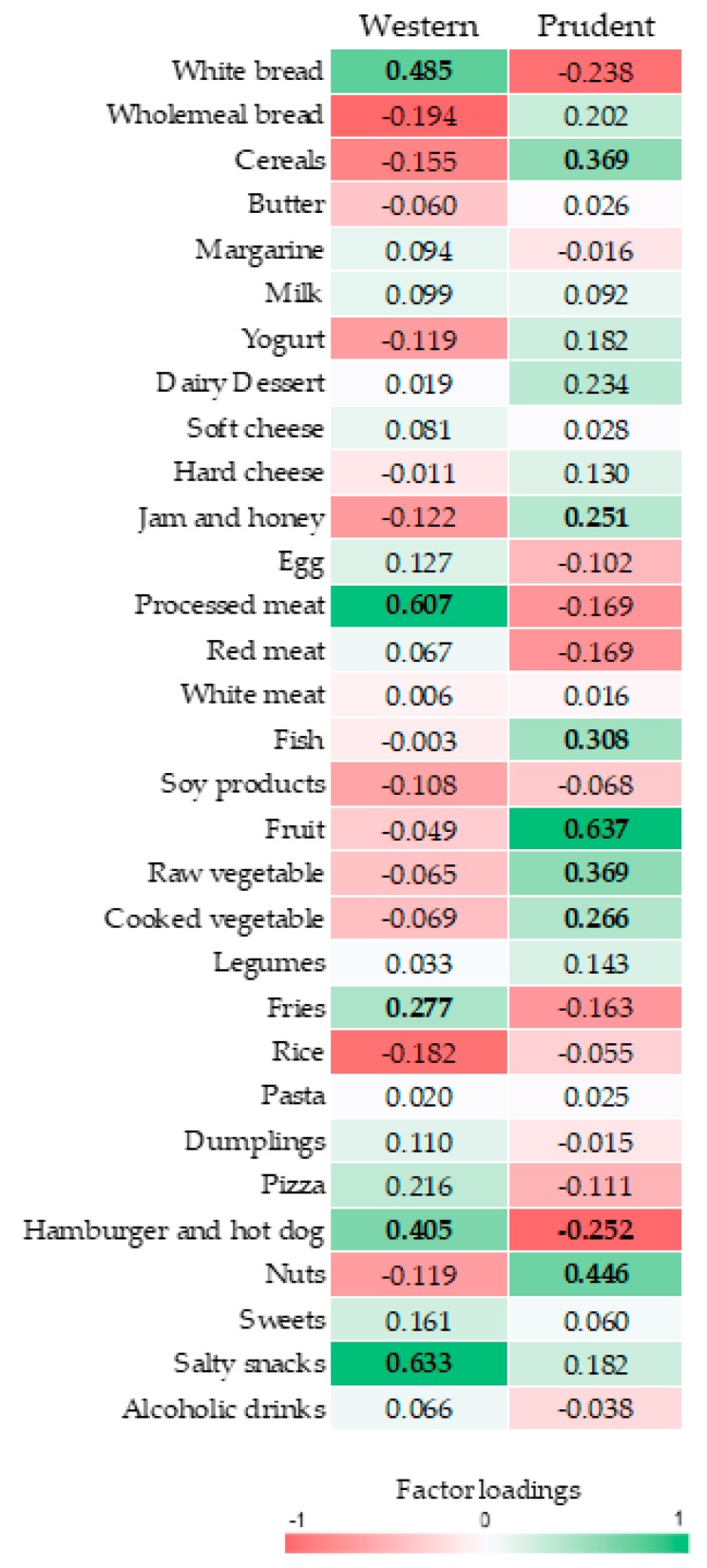
Table of factor loadings characterizing each dietary pattern. Food groups that negatively characterize the dietary patterns are indicated in red. Food groups that positively characterize the dietary patterns are indicated in green. Factor loadings ≥|0.25|are in bold font.

**Figure 3:**
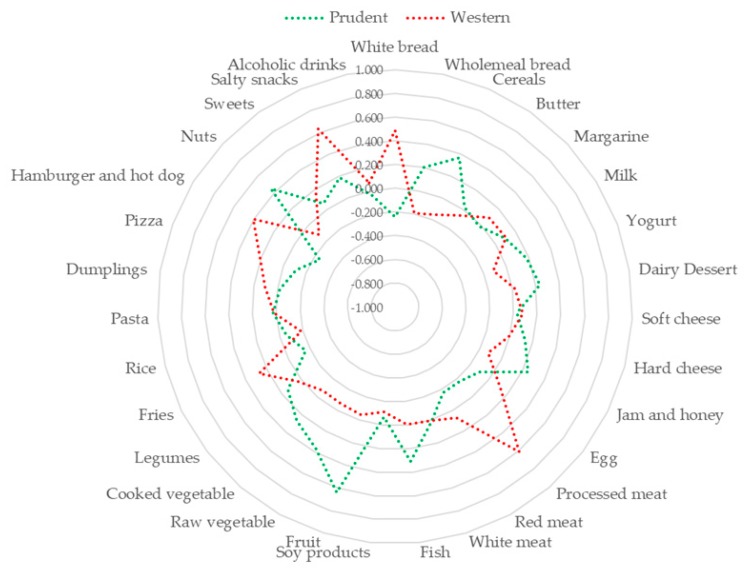
Radar graph of factor loadings characterizing each dietary pattern. Red line indicates factor loadings related to the western dietary pattern. Green line indicates the factor loadings related to the prudent dietary pattern.

Based on the Scree plot (Figure 1), we derived two major dietary patterns with eigenvalues ≥2.0 explaining 13.73% of total variance among 31 food groups. Figure 2 and Figure 3 illustrate factor loadings which characterized each dietary pattern. Accordingly, the first dietary pattern—named western—was positively characterized by high intake of white bread, processed meat, fries, hamburger, hot-dog and salty snacks. By contrast, the second one—named prudent according to the well-accepted term used in this field of research—was positively characterized by high intake of cereals, jam and honey, fish, fruit, raw and cooked vegetables and nuts. Characteristics of study participants by dietary pattern tertiles are reported in Table 2. Particularly, higher adherence to the western dietary pattern was associated with decreasing age and percentage of unemployed, as well as with increasing total energy intake and percentage of male. No statistically significant differences were observed across tertiles of prudent dietary pattern.

### 3.3. The Association of Dietary Patterns with Cardio-Metabolic Parameters

Table 3 shows cardio-metabolic parameters by tertiles of dietary patterns. From T1 to T3 of western dietary pattern, we observed increasing weight, waist circumference, WHR, creatinine, triglycerides and total cholesterol/HDL cholesterol ratio increased, while glycated hemoglobin and HDL cholesterol decreased (*p*-values < 0.05). By contrast, from T1 to T3 of prudent dietary pattern, we observed decreasing weight, BMI, body fat mass, waist and hip circumferences, as well as the prevalence of overweight/obese and abdominal obese subjects (*p*-values < 0.05). In addition, systolic and diastolic BP decreased with increasing tertiles of prudent dietary pattern (*p*-values < 0.05).

### 3.4. The Association of Dietary Patterns with MetS and Its Components

**Figure 4:**
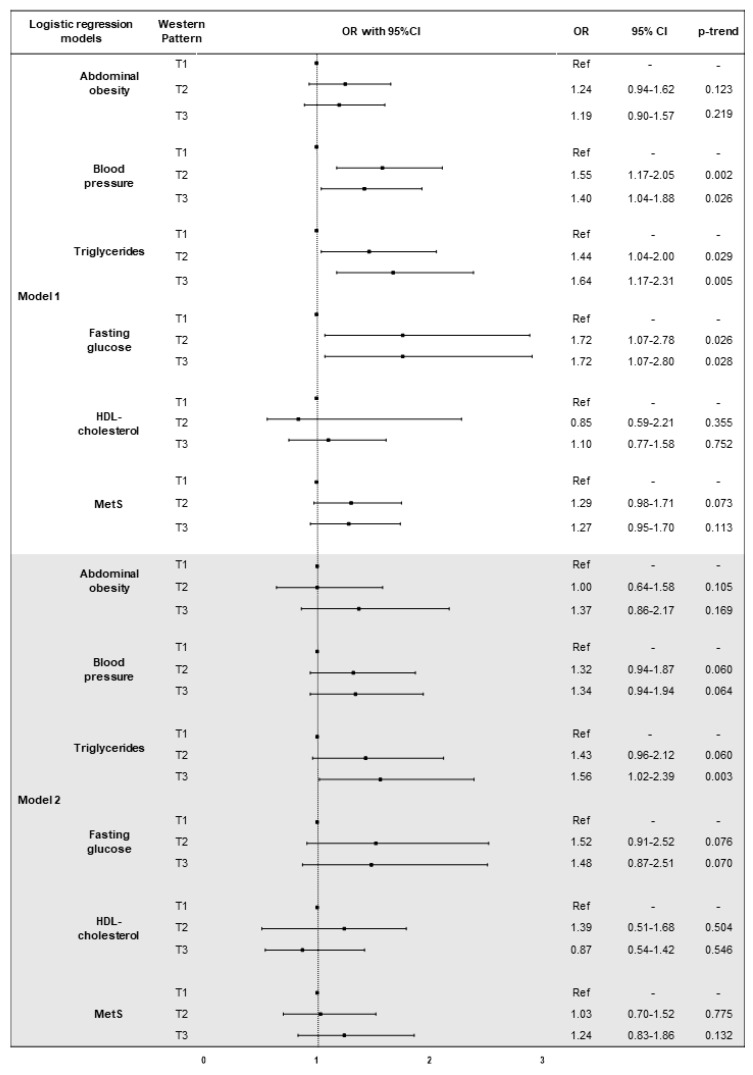
Logistic regression models of the association between western dietary pattern, MetS and it components. Abbreviations: OR, odds ratio; CI, confidence interval; T, tertile; MetS, metabolic syndrome. Model 1: adjusted for age and sex. Model 2: adjusted for age, sex, marital status, employment, educational level, smoking, BMI, total energy intake, and physical activity.

Figure 4 shows results from the logistic regression analysis of the association between western dietary pattern, MetS and its components. After adjustment for age and sex, higher factor score of western dietary pattern was associated with increased odds of abnormal BP, triglycerides and fasting glucose (*p*-trend = 0.009; *p*-trend = 0.005; *p*-trend = 0.015, respectively). The age- and sex-adjusted model also demonstrated that subjects in T2 and in T3 had higher odds of abnormal BP (OR = 1.55, 95%CI = 1.17–2.05, *p* = 0.002; OR = 1.40, 95%CI = 1.04–1.88, *p* = 0.026; respectively), triglycerides (OR = 1.44, 95%CI = 1.04–2.00, *p* = 0.029; OR = 1.64, 95%CI = 1.17–2.31, *p* = 0.005; respectively) and fasting glucose (OR = 1.72, 95%CI = 1.07–2.78, *p* = 0.026; OR = 1.72, 95%CI = 1.07–2.80, *p* = 0.028; respectively), than those in T1. Subjects in T3 also showed higher odds of abnormal triglycerides (OR = 1.56, 95%CI = 1.02–2.39, *p* = 0.003), after adjusting for age, sex, marital status, employment, educational level, smoking, BMI, total energy intake, and physical activity. 

**Figure 5:**
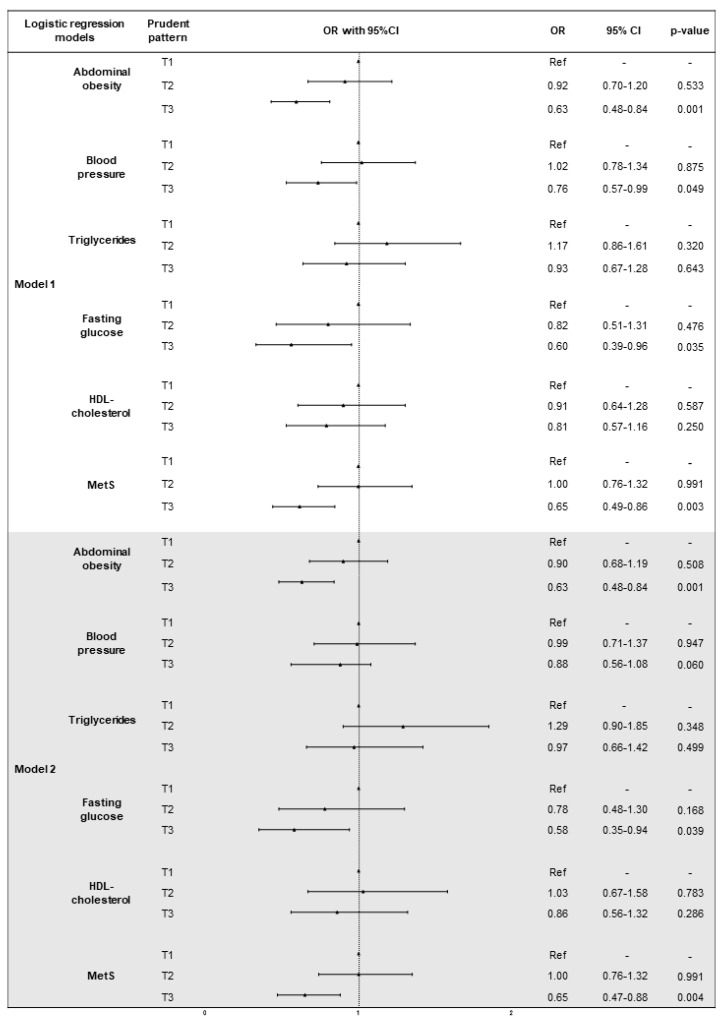
Logistic regression models of the association between prudent dietary pattern, MetS and it components. Abbreviations: OR, odds ratio; CI, confidence interval; T, tertile; MetS, metabolic syndrome. Model 1: adjusted for age and sex. Model 2: adjusted for age, sex, marital status, employment, educational level, smoking, BMI, total energy intake, and physical activity.

Figure 5 shows results from the logistic regression analysis of the association between prudent dietary pattern, MetS and its components. After adjustment for age and sex, higher factor score of prudent dietary pattern was associated with lower odds of abdominal obesity, abnormal BP and MetS (*p*-trend = 0.001; *p*-trend = 0.048; *p*-trend = 0.003, respectively). The age- and sex-adjusted model also showed that subjects in T3 had lower odds of abdominal obesity (OR = 0.63, 95%CI = 0.48–0.83, *p* = 0.001), abnormal BP (OR = 0.76, 95%CI = 0.57–0.99, *p* = 0.049), fasting glucose (OR = 0.60, 95%CI = 0.39–0.96, *p* = 0.035), and MetS (OR = 0.65, 95%CI = 0.49–0.86, *p* = 0.003), than those in T1. In addition, we confirmed that subjects in T3 had lower odds of abdominal obesity (OR = 0.63, 95%CI = 0.48–0.84, *p* = 0.001), abnormal fasting glucose (OR = 0.58, 95%CI = 0.35–0.94, *p* = 0.039), and MetS (OR = 0.65, 95%CI = 0.47–0.88, *p* = 0.004), after adjusting for age, sex, marital status, employment, educational level, smoking, BMI, total energy intake, and physical activity. 

## 4. Discussion

In the current study, according to the IDF definition [5], we reported that prevalence of MetS was 38.2% in a randomly-selected sample of the Czech urban population, which is similar with that reported among Polish, neighboring Central European population [25]. The diagnosis of MetS was more common among male, low-educated, unemployed and smoking subjects. Moreover, it was observed higher prevalence of MetS with decreasing physical activity level. To evaluate the association with MetS, we first derived two a posteriori dietary patterns which characterized the dietary habits of the study population. The first pattern—named “western”—was characterized by high intake of white bread, processed meat, fries, hamburger, hot-dog and salty snacks; the second one—named “prudent”—consisted of high intake of cereals, jam and honey, fish, fruit, raw and cooked vegetables, and nuts. Foods composition of these dietary patterns partially overlapped with those examined by two previous meta-analyses [8,9]. 

Consistently with results from the Polish population [25], we demonstrated the association of western dietary pattern with some of the individual MetS components, but not with overall MetS risk. Although a previous meta-analysis, comparing the highest to the lowest categories of western/unhealthy dietary patterns, reported a pooled OR for MetS of 1.28 (95%CI = 1.17–1.40), results varied across studies [8]. This inconsistency could be attributed to differences in socio-demographic characteristics, ethnicity, behavioral and lifestyle factors. In the present study we observed that weight, waist circumference and WHR increased with increasing adherence to western dietary pattern, but we failed to replicate previous evidence showing that unhealthy dietary habits may increase the risk of abdominal obesity [26,27,28,29]. Compared to low adherence, medium-high adherence to the western dietary pattern was associated with increased odds of abnormal BP, triglycerides and fasting glucose, after adjusting for age and sex. This is consistent with several lines of evidence demonstrating that western dietary patterns were associated with levels of blood pressure and serum lipids [30,31,32]. By contrast, other studies reported that dietary habits, characterized by high consumption of meat, affected HDL levels but not blood pressure, LDL and triglycerides levels [25,33]. However, we confirmed a robust positive association between adherence to western dietary pattern and abnormal triglycerides (OR = 1.56, 95%CI = 1.02–2.39), by adding to the regression model both socio-demographic (i.e., marital status, employment and educational level) and behavioral factors (i.e., smoking, BMI, total energy intake and physical activity). Since MetS and NAFLD predict the same metabolic risk profile, then it follows that the two disorders should share similar pathophysiological features [6]. In fact, a similar dietary pattern rich in animal food was also associated with the risk of fatty liver disease in general [34], and NAFLD in particular [35]. 

By contrast, we demonstrated lower odds of MetS (OR = 0.65, 95%CI = 0.47–0.88) among participants with high adherence to prudent dietary pattern, after adjusting for age, sex, marital status, employment, educational level, smoking, BMI, total energy intake, and physical activity. This is consistent with results from the abovementioned meta-analysis showing a pooled OR of 0.83 (95%CI = 0.76–0.90) [8]. Accordingly, our results reported decreasing blood pressure and prevalence of abdominal obesity with increasing adherence to prudent dietary pattern. In fact, the age- and sex-adjusted model showed that subjects with high adherence had lower odds of abdominal obesity (OR = 0.63, 95%CI = 0.48–0.83), as well as abnormal BP (OR = 0.76, 95%CI = 0.57–0.99) and fasting glucose (OR = 0.60, 95%CI = 0.39–0.96), than those with low adherence. In addition, we established a robust association of adherence to prudent dietary pattern with abdominal obesity (OR = 0.63, 95%CI = 0.48–0.84) and abnormal fasting glucose (OR = 0.58, 95%CI = 0.35–0.94), by adding to the regression model both socio-demographic (i.e., marital status, employment and educational level) and behavioral factors (i.e., smoking, BMI, total energy intake and physical activity). Our results are in line with previous studies demonstrating the protective effect of healthy dietary patterns against the risk of central obesity [27,36,37,38], impaired fasting glucose [39], insulin resistance [40], and diabetes [41]. All these pathological features are also related to NAFLD and it has been similarly proved that adherence to a prudent dietary pattern reduced the risk of NAFLD [42]. 

A weakness of our study includes the cross-sectional design, which does not allow demonstrating the causality of the relationships. In fact, it is possible that the diagnosis of metabolic disorders may influence dietary habits. Moreover, the effect of unmeasured socio-demographic factors (e.g., income, food security, food access and food availability) physiological condition (e.g., menopause status) and comorbidities cannot be completely excluded. We estimated food intakes using a FFQ with standard portion sizes, which does not preclude measurement error and may suffer from inaccuracies. Nevertheless, standard portion sizes were obtained from an individual dietary survey on the national level, which involved age and gender representative sample of the Czech population [22]. Accordingly, the food compositions of our PCA-derived dietary patterns were consistent with those identified by previous studies across different European populations [11,25,28,43,44]. 

Our study has also several strengths, including the large sample that was randomly selected from the urban population of Brno (Czech Republic). Therefore, these results, obtained from a nationally representative sample, can be extrapolated to the general Czech population. Moreover, cardio-metabolic parameters and anthropometric measures were assessed using standard and validated protocols, allowing comparisons with other well-designed studies. To this purpose, we classified study participants according to the IDF definition [5], which provides one of the most widely used classifications of MetS. Finally, the majority of our results are robust, since they have been confirmed after adjusting for several socio-demographic and behavioral factors. 

## 5. Conclusions

To our knowledge, the present study is the first investigating the association of dietary patterns with MetS in a random large sample of Czech urban population. Whilst we confirmed the deleterious effect of a western dietary pattern on several metabolic risk factors, our results also indicated that the consumption of a diet rich in cereals, fish, fruit and vegetables is associated with a healthier metabolic profile and lower risk of MetS. Although additional prospective research should be conducted, public health professionals could benefit from these findings, developing and validating novel potential preventive strategies against MetS and its complications.

## Figures and Tables

**Table 1:** Characteristics of study population and comparison between MetS patients and controls.

Characteristics	Total (*N* = 1934)	MetS (*N* = 739)	Control (*N* = 1195)	*p*-Value ^a^
Age, years ^b^	47.0 (19.0)	54.0 (15.0)	41.5 (17.0)	**<0.001**
Sex (% male)	44.6%	48.6%	42.2%	**0.017**
Marital status (% married)	61.7%	69.1%	58.3%	**<0001**
Employment (% unemployed)	18.1%	25.7%	13.3%	**<0.001**
Smoking (% current smokers)	23.7%	26.7%	22.1%	**0.041**
Total energy intake, kcal ^b^	2006 (916)	1963 (912)	2045 (910)	**0.013**
Physical activity, MET min/week ^b^	3175 (4782)	2994 (4388)	3252 (4340)	**0.009**
Hypertension	34.6%	66.0%	18.2%	**<0.001**
Use of antihypertensives	17.0%	36.5%	5.2%	**<0.001**
Hyperlipidemia	65.4%	84.3%	54.0	**<0.001**
Use of hypolipidemics	5.4%	11.2%	1.9%	**<0.001**
Weight, kg ^b^	75.7 (23.0)	88.3 (20.2)	69.8 (17.9)	
BMI, kg/m^2 b^	24.8 (5.8)	28.6 (5.3)	23.1 (3.9)	**<0.001**
BMI categories (%)				
Underweight	2.9%	0%	4.6%	**<0.001**
Normal weight	48.5%	14.7%	69.3%	
Overweight	33.6%	50.5%	23.1%	
Obese	15.1%	34.8%	3.0%	
Waist circumference ^b^	87.0 (20.0)	100.0 (13.0)	81.0 (14.0)	**<0.001**
Hip circumference ^b^	102.0 (10.0)	107.0 (10.0)	99.0 (8.0)	**<0.001**
WHR ^b^	0.85 (0.14)	0.92 (0.12)	0.81 (0.12)	**<0.001**
Abdominal Obesity (%)	52.2%	100%	22.6%	**<0.001**
Body Fat Mass ^b^	18.4 (12.1)	25.5 (11.1)	14.6 (8.0)	**<0.001**
Systolic Blood Pressure, mmHg ^b^	117.0 (19.5)	127.5 (19.8)	112.5 (14.0)	**<0.001**
Diastolic Blood Pressure, mmHg ^b^	79.0 (13.0)	85.5 (11.3)	76.3 (10.5)	**<0.001**
Glycated Haemoglobin, nmol/mol ^b^	39.0 (6.0)	41.0 (5.0)	38.0 (5.0)	**<0.001**
Fasting Glucose, nmol/L ^b^	4.8 (0.7)	5.1 (0.7)	4.7 (0.6)	**<0.001**
Creatinine, nmol/L ^b^	75.0 (18.0)	76.0 (18.5)	75.0 (18.0)	**0.312**
Triglycerides, nmol/L ^b^	1.0 (0.8)	1.4 (0.9)	0.8 (0.5)	**<0.001**
Total Cholesterol, nmol/L ^b^	5.1 (1.3)	5.4 (1.4)	4.9 (1.3)	**<0.001**
HDL Cholesterol, nmol/L ^b^	1.5 (0.5)	1.3 (0.5)	1.6 (0.5)	**<0.001**
LDL Cholesterol, nmol/L ^b^	3.0 (1.2)	3.3 (1.3)	2.9 (1.2)	**<0.001**
Total Cholesterol/HDL-ratio ^b^	**3.3 (1.4)**	**4.0 (1.5)**	**3.0 (1.1)**	**<0.001**

Abbreviations: IQR Interquartile range; MET Metabolic Equivalent of Task; BMI body mass index; WHR waist-to-hip ratio. ^a^ Statistically significant *p*-values (*p* < 0.05) are indicated in bold font. ^b^ Data reported as median (IQR).

**Table 2:** Characteristics of study participants by tertiles of dietary patterns.

Characteristics	Western Dietary Pattern				Prudent Dietary Pattern			
	T1 (*N* = 639)	T2 (*N* = 651)	T3 (*N* = 644)	*p*-Value ^a^	T1 (*N* = 646)	T2 (*N* = 633)	T3 (*N* = 655)	*p*-Value ^a^
Age, years ^b^	50.0 (19.0)	48.0 (18.0)	43.0 (18.0)	**<0.001**	47.0 (19.0)	48.0 (19.0)	46.0 (20.0)	0.215
Sex, (% male)	32.9%	43.3%	56.5%	**<0.001**	44.3%	45.2%	43.4%	0.805
Marital status, (% married)	36.2%	38.3%	40.4%	0.298	38.2%	37.5%	39.3%	0.807
Employment, (% unemployed)	21.7%	17.4%	14.8%	**0.006**	16.3%	17.6%	20.0%	0.224
Smoking, (% current smokers)	20.7%	23.4%	27.2%	0.058	26.2%	22.2%	22.8%	0.275
Total energy intake, kcal ^b^	1795.2 (873.4)	2000.2 (909.0)	2214.6 (969.8)	**<0.001**	1992.9 (925.7)	1993.5 (913.4)	2031.3 (943.7)	0.334
Physical activity, MET-min/week ^b^	3644.5 (4310.2)	3120.0 (4909.1)	3338.0 (5235.0)	0.325	3338.0 (5408.3)	3456.5 (5281.1)	3300.0 (4292.6)	0.796
Hypertension	34.2%	39.2%	35.7%	0.244	38.6%	37.6%	32.9%	0.129
Use of antihypertensives	17.0%	18.7%	15.1%	0.323	17.2%	19.2%	14.5%	0.145
Hyperlipidemia	33.9%	32.4%	37.4%	0.253	35.1%	37.9%	30.6%	0.059
Use of hypolipidemics	6.1%	4.5%	5.5%	0.533	5.9%	5.3%	4.9%	0.791

Abbreviations: T tertile; IQR Interquartile range; MET Metabolic Equivalent of Task. ^a^ Statistically significant *p*-values (*p* < 0.05) are indicated in bold font. ^b^ Data reported as median (IQR).

**Table 3:** Cardio-metabolic parameters of study participants by tertiles of dietary patterns.

Characteristics	Western Dietary Pattern				Prudent Dietary Pattern			
	T1 (*N* = 639)	T2 (*N* = 651)	T3 (*N* = 644)	*p*-Value ^a^	T1 (*N* = 646)	T2 (*N* = 633)	T3 (*N* = 655)	*p*-Value ^a^
Weight, kg ^b^	72.4 (19.8)	78.2 (24.0)	77.5 (23.7)	**0.002**	77.2 (22.5)	76.6 (24.7)	74.1 (22.1)	**0.006**
BMI, kg/m^2 b^	24.5 (5.2)	25.2 (6.2)	24.8 (5.9)	0.128	25.1 (6.0)	25.2 (5.7)	24.3 (5.4)	**<0.001**
BMI categories (%)								
Underweight	3.9%	2.5%	2.3%	0.425	2.5%	1.6%	4.5%	**0.005**
Normal weight	49.7%	45.8%	48.9%		45.9%	45.9%	52.5%	
Overweight	32.8%	34.7%	34.0%		34.6%	35.6%	31.4%	
Obese	13.6%	17.0%	14.9%		17.1%	16.9%	11.6%	
Waist circumference ^b^	85.0 (18.0)	87.5 (21.0)	88.0 (21.0)	**0.001**	88.0 (19.8)	87.0 (20.0)	85.0 (18.0)	**0.001**
Hip circumference ^b^	102.0 (11.0)	102.0 (10.8)	101.0 (10.0)	0.236	102.0 (10.0)	103.0 (11.0)	101.0 (9.0)	**0.001**
WHR ^b^	0.83 (0.13)	0.85 (0.14)	0.87 (0.14)	**<0.001**	0.86 (0.14)	0.85 (0.13)	0.84 (0.15)	0.075
Abdominal Obesity (%)	52.8%	54.6%	50.3%	0.401	56.3%	55.9%	45.8%	**0.001**
Body Fat Mass ^b^	18.4 (12.4)	18.6 (13.0)	17.5 (11.4)	0.121	19.3 (12.7)	18.7 (12.2)	16.9 (10.8)	**<0.001**
Systolic Blood Pressure, mmHg ^b^	116.5 (19.5)	117.5 (18.0)	116.5 (19.5)	0.341	117.5 (19.5)	117.5 (20.3)	115.5 (18.5)	**0.022**
Diastolic Blood Pressure, mmHg ^b^	78.3 (12.5)	80.0 (13.0)	79.0 (14.0)	0.087	79.5 (13.5)	80.0 (13.3)	78.0 (12.0)	**0.021**
Glycated Haemoglobin, nmol/mol ^b^	40.0 (5.0)	39.0 (5.0)	39.0 (5.0)	**0.002**	39.0 (6.0)	39.0 (5.0)	39.0 (6.0)	0.279
Fasting Glucose, nmol/L ^b^	4.8 (0.6)	4.9 (0.7)	4.8 (0.6)	0.187	4.8 (0.7)	4.9 (0.7)	4.8 (0.7)	0.157
Creatinine, nmol/L ^b^	73.0 (17.3)	75.0 (18.8)	78.0 (18.0)	**<0.001**	75.0 (19.0)	76.0 (17.5)	74.0 (18.0)	0.281
Triglycerides, nmol/L ^b^	0.9 (0.7)	1.0 (0.9)	1.1 (0.8)	**0.001**	1.0 (0.7)	1.0 (0.8)	1.0 (0.9)	0.278
Total Cholesterol, nmol/L ^b^	5.1 (1.4)	5.2 (1.4)	5.1 (1.3)	0.061	5.1 (1.3)	5.2 (1.3)	5.1 (1.3)	0.687
HDL Cholesterol, nmol/L ^b^	1.6 (0.5)	1.5 (0.5)	1.4 (0.5)	**<0.001**	1.5 (0.5)	1.5 (0.5)	1.5 (0.5)	0.206
LDL Cholesterol, nmol/L ^b^	3.1 (1.3)	3.0 (1.2)	3.0 (1.2)	0.388	3.0 (1.2)	3.1 (1.2)	3.0 (1.3)	0.761
Total Cholesterol/HDL-ratio ^b^	3.2 (1.3)	3.3 (1.4)	3.4 (1.6)	**0.001**	3.3 (1.4)	3.3 (1.4)	3.3 (1.4)	0.578

Abbreviations: T tertile; IQR Interquartile range. BMI body mass index; WHR waist-to-hip ratio. ^a^ Statistically significant *p*-values (*p* < 0.05) are indicated in bold font. ^b^ Data reported as median (IQR).
